# Exploring efficacy of spiritual-based interventions (SBIs) in addressing depressive symptoms among cardiac patients in MENA region: a scoping review

**DOI:** 10.3389/fpsyg.2025.1552678

**Published:** 2025-04-08

**Authors:** Rizwana Amin

**Affiliations:** Psychology Department, Effat University, Jeddah, Saudia Arabia

**Keywords:** spiritual-based interventions, depressive symptoms, cardiac patients, MENA region, scoping review

## Abstract

**Background:**

Depression is a widespread psychological issue among patients with cardiac diseases, which affects treatment adherence, recovery, and overall quality of life. Spiritual-based interventions (SBIs) have gained attention for their potential to alleviate depressive symptoms. However, there is a dearth of research investigating the efficacy of these interventions in the Middle East and North Africa (MENA) region.

**Objective:**

This scoping review aims to synthesize available evidence on the efficacy of spiritual-based interventions in reducing depressive symptoms among cardiac patients in the MENA region.

**Methods:**

The present research used a systematic approach to searching electronic databases such as SCOPUS, Web of Science, and ESBECOhost Arab research world in the English language from 2015 to 2025, based on the Arksey and O'Malley framework. Papers were identified based on spiritual-based Intervention addressing depressive symptoms among cardiac patients in the MENA region. Studies were analyzed using the Population-Concept-Context (PCC) framework, as outlined in the Preferred Reporting Items for Systematic Reviews and Meta-Analyses for Scoping Reviews (PRISMA-ScR).

**Results:**

The analysis pinpoints numerous Spiritual Interventions, such as prayer, mindfulness, and Faith-based counseling, as usual practices. The results showed that SBIs are related to a decrease in depression symptomology and enhancement of psychological wellbeing. Nevertheless, variability in the study models, small amounts of samples, and sparse long-term studies are also some of the current study's limitations.

**Conclusion:**

This research indicates that the efficacy of spiritual-based interventions can reduce depressive signs in cardiac patients in the MENA region. However, additional research is required to ascertain long-term efficacy and cross-cultural effectiveness.

## 1 Introduction

Cardiovascular diseases (CVDs) represent a predominant source of morbidity and mortality globally, resulting in approximately 17.9 million deaths per year (WHO, 2021). These diseases place a heavy burden in the Middle East and North Africa (MENA) region, with urbanization, lifestyle changes, and socioeconomic disparities fueling the rise in coronary artery disease, myocardial infarction, and heart failure (Alhuneafat et al., 2024). There are many ongoing medical progress for more accurate diagnosis and treatment of cardiac diseases, increasingly, it is being recognized that both physical and mental health play crucial roles in shaping patient outcomes (Borkowski and Borkowska, 2024). Depression constitutes one of the most common and important psychological problems in cardiac patients with negative effects on the quality of life and worsening of such diseases (Li et al., 2023). Cardiac disease has a high prevalence of depression among individuals with studies estimating occurrence in 20%−40% of patients (Li et al., 2023; Moradi et al., 2022).

The relationship between cardiac diseases and depression is bidirectional (Ogunmoroti et al., 2022). Cardiac disease increases the risk of developing depressive symptoms because of chronic stress, functional limitation, and the psychological effects related to a life-threatening diagnosis (Berk et al., 2023) while depression hardly influences cardiac outcomes through pathways such as increased inflammation, impaired autonomic regulation, and non-adherence (Li et al., 2023). Cardiac patients who exhibit depressive symptoms experience higher rehospitalization rates (Veskovic et al., 2023), decreased adherence to medical instructions, and greater mortality rates (Berk et al., 2023). With these profound consequences, the treatment of depression in cardiac patients is a key element of comprehensive cardiovascular care (Ullah et al., 2023). Over the past few years, there has been increasing evidence about the role of spirituality and religion in enhancing mental wellbeing (Zarzycka and Puchalska-Wasyl, 2019).

Spirituality is a wide concept related to beliefs, practices, and experiences that give individuals a sense of meaning as well as purpose along with feelings of connection to something greater than themselves (de Brito Sena et al., 2021). In psychological interventions, spirituality may include prayer, meditation, mindfulness, and involvement in religious texts or community. Adhering to these practices can provide optimism and support, especially for those experiencing serious ailments such as heart disease (Currier et al., 2023). Numerous studies substantiate the link between spirituality and mental health (Ghuloum et al., 2024). Research now supports the fact that spirituality itself can help decrease symptoms of depression and anxiety, increase the ability for emotional coping, and even improve overall quality of life (Nuraeni et al., 2024). Hence, its advantages can be attributed to cultivating hope to understand and accept adversities and creating a supportive environment (Haufe et al., 2024).

Furthermore, spiritual interventions reflect people's cultural and religious practices worldwide, especially within the MENA region where spirituality is ingrained in daily life (Ballout, 2024; Fenkl and Larry, 2024). The MENA region is known for its diverse cultures and religious backgrounds where the predominant faith practiced is Islam. The concepts of Spirituality and health are closely intertwined in Islamic teachings and accentuate the priority of wellbeing by inculcating gratitude and tawakkal (trust in Allah). People in the region, practice salat (prayer), recitation of the Holy Quran, and Dhikr which is regarded as a source of comfort and healing during adversities (Djebbi, 2022). Although spirituality has been prominent in the MENA cultural context, relatively few studies have examined its use in the Psychological Treatment of cardiac patients. Unfortunately, most studies focused on spiritual-based psychological interventions have been conducted in Western cultures where spirituality differs from what is seen in Muslim countries and may not be a major defining component of peoples' lives (Rogers, 2023). These studies showed promising effects in reducing distress and enhancing wellbeing (Hatala and Kerstin, 2023; Tönis et al., 2023).

Consequently, the present study proposes an area of research that is currently unexplored in the literature on spiritual interventions focusing on the acceptability and efficacy of interventions for spiritual care for the people of the MENA region based on their culture and beliefs. Although the potential benefits of spiritual-oriented psychological interventions are encouraging, there is still a significant deficit in comprehensive evidence regarding their application and effectiveness among cardiac patients in the MENA region. Therefore, a scoping review presents an ideal methodological approach to fill this gap by systematically evaluating the breadth and depth of existing literature, identifying key themes, and pinpointing areas for future research. The present review aims to answer the following key questions:

1. What spiritual-based interventions are being utilized for cardiac patients in the MENA region?
2. What improvements have been observed in depressive symptoms and other outcomes?
3. What cultural and contextual factors could affect the use and efficacy of the interventions?

## 2 Methods

### 2.1 Research design

The present study follows a scoping review framework (Arksey and O'Malley, 2005) to explore the breadth of available evidence on spiritual-based psychological interventions and their impact on depressive symptoms in cardiac patients within the MENA region. The studies are analyzed through the Population-Concept-Context (PCC) framework, as outlined in the Preferred Reporting Items for Systematic Reviews and Meta-Analyses for Scoping Reviews[(PRISMA-ScR), (Arksey and O'Malley, 2005)]. In this study, PCC is explained as the population which refers to Cardiac patients experiencing depressive symptoms, the concept denotes Spiritual-based psychological interventions (e.g., prayer, mindfulness, and religious counseling), and Context represents Studies conducted in the MENA region.

### 2.2 Eligibility criteria

Studies included in this review involve cardiac patients with depressive symptoms particularly, those undergoing psychological intervention based on spirituality or religiosity within the MENA region. Moreover, peer-reviewed quantitative articles using experimental design published in English from January 2015 to December 2024 have been included. Whereas, cross-sectional studies, review papers, editorials, conference proceedings, book chapters, qualitative, mixed methods studies, and gray literature have been excluded. Additionally, studies that do not employ spiritual/religious interventions for the treatment of depression among cardiac patients and conducted outside the MENA region.

### 2.3 Search strategy

A comprehensive literature review was conducted by exploring multiple databases including PubMed, Scopus, Web of Science, and EBSCOhost-Arab World Research Source. Databases were searched using the terms “spiritual-based Interventions” OR “religious interventions” OR “faith-based interventions” AND “depression” OR “depressive symptoms” OR “post-cardiac depression” AND “cardiac Patients” OR “cardiovascular diseases” OR “coronary artery diseases” OR “heart Failure” OR “CABG” AND “MENA region” OR “Middle East” OR “North Africa.” The terms “review” and “meta” were excluded from the search results. Duplicates were removed. The abstracts from all the results of this search criteria were then read and sorted to verify whether they met the inclusion or exclusion criteria. The papers were then further read in their entirety before once more repeating the process of verifying eligibility.

### 2.4 Study selection

**Figure 1:**
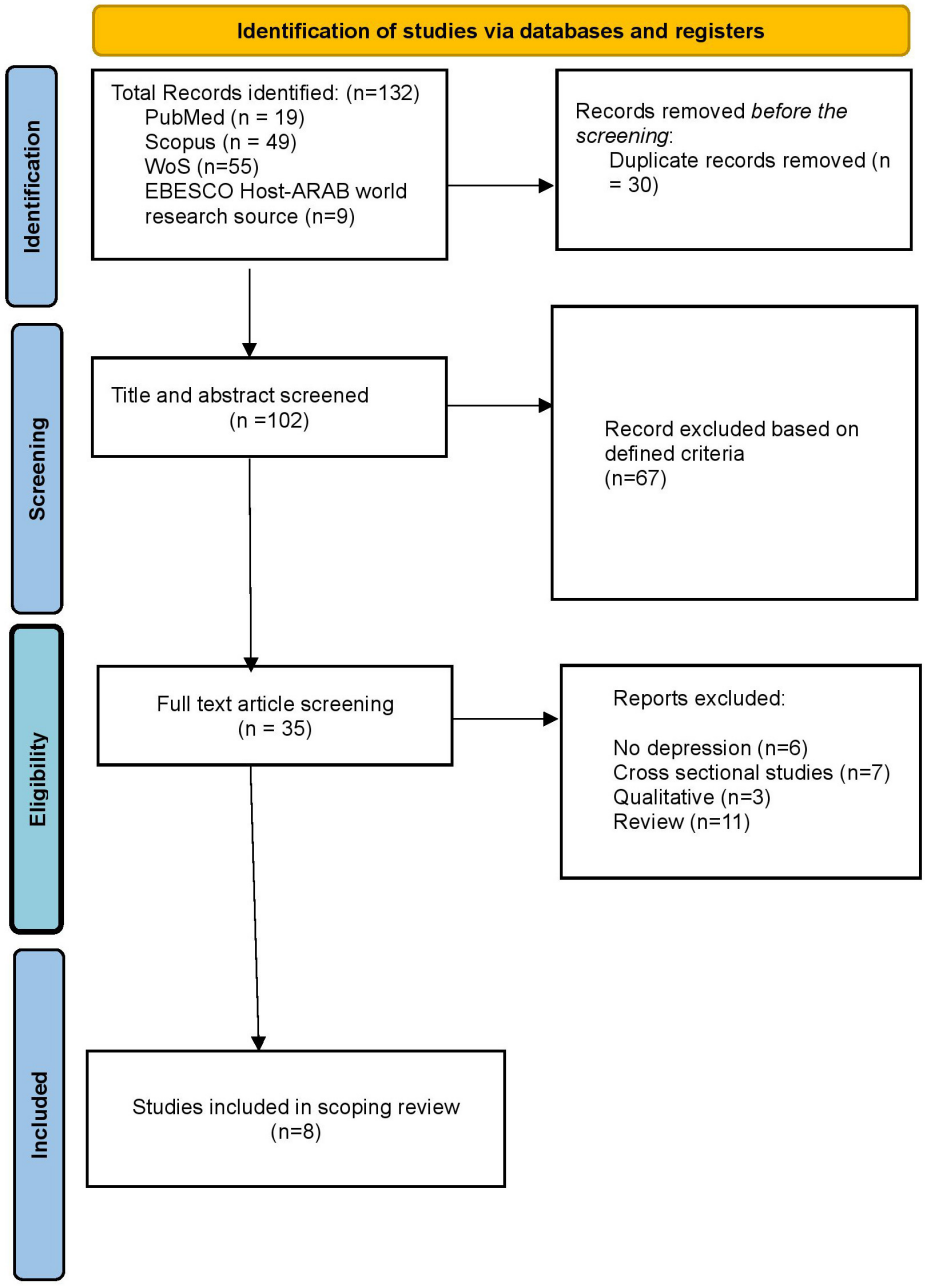
Study selection flow chart from Tricco et al. (2018).

The database search started with the identification of relevant studies. A total of 132 studies were identified and initially removed 30 duplications. Consequently, 102 remaining studies have been screened based on title and abstract relevance resulting exclusion of 67 studies. Within the screening process, 35 studies were screened on defined inclusion and exclusion criteria. Only 8 studies completely fulfilled the inclusion criteria. The screening process and search strategy using the PRISMA guidelines are indicated in Figure 1.

### 2.5 Data extraction

Extracted data highlights essential characteristics such as authors, publication year, country, setting, research aim, design sample, measures, interventions, duration and number of sessions, and the outcome.

### 2.6 Quality assessment

The present review was evaluated through the JBI critical appraisal checklist for randomized control trials (RCTs) and quasi-experiments. The JBI Critical Appraisal Checklist for Randomized Control Trials (RCTs) (Barker et al., 2023) assesses study validity through the evaluation of randomization, allocation concealment, blinding, follow-up, outcome measures, and statistical analysis. Similarly, the JBI Checklist for Quasi-experimental Studies (Barker et al., 2024) emphasizes intervention clarity, group comparability, outcome measurement, and confounding factors. Both approaches rigorously assess the methodological strengths and limitations, ensuring the inclusion of high-quality research. While scoping reviews typically map existing literature without a critical appraisal, integrating these tools enhances transparency, highlights study robustness, and identifies areas needing further investigation, strengthening the review's overall credibility.

### 2.7 Data analysis strategy

The Present study identified, extracted and interpreted key themes from the reviewed papers using thematic analysis based on the framework established by Braun and Clarke (2006). This process involved iterative steps that combined both inductive and deductive coding. Table 1 represents themes, codes, types, and description.

**Table 1:** Themes, codes, types, and description.

**Themes**	**Coding of themes**	**Type of codes**	**Descriptions of codes**
Culturally tailored interventions as therapeutic tools	Culturally adapted psychotherapy, Traditional healing integration, and Community-based mental health programs	Deductive	Studies highlight culturally responsive interventions that incorporate traditional and community-based approaches for improving mental health (e.g., Abdi et al., 2019; Nikrahan et al., 2016).
Reduction in depression and anxiety	Decrease in depressive symptoms, Anxiety reduction, Emotional stability	Inductive	Studies report a decline in clinical depression and anxiety levels among participants following tailored interventions (e.g., AbuRuz et al., 2023; Amjadian et al., 2020).
Enhancement of positive psychological states	Psychological resilience, Increased life satisfaction, Greater emotional wellbeing	Inductive	Interventions led to improved resilience, optimism, and overall psychological wellbeing (e.g., Ehsan et al., 2019; Moghadam et al., 2021).
Cultural beliefs and societal norms	Community, collectivistic society, Gender roles, religious beliefs, family dynamics, Interpersonal relationship	Inductive	Traditional gender norms influence mental health treatment accessibility and intervention effectiveness (Masjedi-Arani et al., 2020; Tajbakhsh et al., 2018); amily support can enhance adherence to treatment (AbuRuz et al., 2023); community-based interventions are often more successful than individual-focused approaches, as social networks provide emotional and practical support for adherence and long-term impact (Moghadam et al., 2021; Nikrahan et al., 2016).
Holistic health improvements	Physical wellbeing, Mind-body connection, Lifestyle modifications	Deductive	Studies emphasize how psychological interventions also promote physical health improvements, such as reduced stress levels and healthier lifestyle choices (e.g., Masjedi-Arani et al., 2020; Tajbakhsh et al., 2018).
Mechanism of change	Cognitive restructuring, Emotional regulation, Therapeutic engagement	Deductive	The process of change is linked to shifts in cognition, emotion regulation strategies, and sustained therapeutic engagement (e.g., Nikrahan et al., 2016; Moghadam et al., 2021).

Moreover, the study summarized participant characteristics through data aggregation. This approach systematically identifies themes and gain a clearer understanding of the impact of culturally adapted therapies on mental health.

## 3 Results

A total of eight peer-reviewed articles were included in this study. All articles utilize Randomized control trials or quasi-experiments to assess the effectiveness of spiritual-based interventions in reducing depression among cardiac patients within the MENA region.

### 3.1 Characteristics of studies

Among eight studies, the earliest study was published in 2016, and the latest one in 2023. The total sample size for all studies was 651 patients with heart diseases. Seven were conducted in Iran whereas one was in Jordan. The research design for the six studies was RCT and the remaining employed quasi-experiment. All the studies have been carried out in hospital settings.

### 3.2 Participant characteristics

An analysis of patients with cardiac diseases revealed that 184 were diagnosed with heart failure (HF), 422 underwent coronary artery bypass grafting (CABG), and 45 had coronary artery disease (CAD). Participants' ages ranged from 32 to 75 years. Participants were randomly assigned in six studies into experimental/intervention (*n* = 244) and control group (238) and only two studies participants were equally allocated to intervention (*n* = 90) and control group (*n* = 70). In all studies, depression was assessed through self-report inventories (DASS, BDI, HADS, IHF-QOL subscale for psychological conditions). Out of eight, four studies clearly describe the number of female (*n* = 166) and male (*n* = 255) participants. Table 2 indicates the summary of study characteristics.

**Table 2:** Studies and participants' characteristics.

**Researcher**	**Year**	**Paper title**	**Country**	**Setting**	**Aim**	**Design**	**Sample**	**Measures**	**Duration and # of Sessions**	**Intervention**	**Outcome**
Abdi et al.	2019	The Effect of Religion Intervention on Life Satisfaction and Depression in Elderly with Heart Failure.	Iran	Hospital	The current study was conducted to determine the effect of religious intervention on life satisfaction and depression in the elderly with heart failure	Quasi- experimental study	100 Elderly with Heart Failure	1. Life satisfaction questionnaire of LSI-Z (Wood et al., 1969)2. Beck depression inventory (Beck et al., 1996)	6 weeks [six educational sessions each in a week and lasting about 30–45 min]	Spiritual religious strategies (Reading verses from the Holy Quran, offering prayers, participating in spiritual- religious programs, Practicing forgiveness, and finally)	The positive effect of a religion- spiritual program on depression in patients with heart failure.
AbuRuz et al.	2023	The Effect of Holy Quran Audio Therapy on Depression and Anxiety Among Jordanian Patients Following CABG: A Randomized Control Trial	Jordan	Hospital	To examine the effect of Holy Quran audio therapy on anxiety and depression among Arabic- speaking post- CABG patients.	Randomized controlled trial	165 post- CABG ICU patients	Depression Anxiety Stress Scale (Lovibond and Lovibond, 1995).	Ten min over two sessions on 2 days, one after the other	Holy Quran audio therapy (Surah Al- Rahman)	Holy Quran audio therapy was linked to statistically significant positive effects.
Amjadian et al.	2020	A pilot randomized controlled trial to assess the effect of Islamic spiritual intervention and breathing technique with heart rate variability feedback on anxiety, depression, and psycho-physiologic coherence in patients after coronary arterybypass surgery	Iran	Hospital	The study investigated the effects of Islamic religious and breathing techniques on depression in coronary artery bypass graft surgery (CABG) patients	Randomized controlled trial	60 Patients with CABG	Depression Anxiety Stress Scale (Lovibond and Lovibond, 1995)	8 weeks [8 sessions each in a week]-2 hour	Religious-based therapy Islamic teachings (Psychoeducation about spirituality and religion).	The findings showed that depression was reduced more in the religious intervention group.
Tajbakhsh et al.	2018	The Effect of Spiritual Care on Depression in Patients Following Coronary Artery Bypass Surgery:A Randomized Controlled Trial	Iran	Hospital	This study aimed to determine the effect of a nurse- delivered spiritual care intervention on depression following coronary artery bypass graft (CABG) surgery.	Randomized controlled trial	64Iranian Iranian patients undergoing CABG	Depression Anxiety Stress Scale (Lovibond and Lovibond, 1995)	5-week [each session in a week]	Spiritual- religious strategies [Reading sacred books (The Holy Quran), prayer, expression of a religious patterns story, participating in religious– spiritual programs, repentance and forgiveness, and intellectual analysis ofethical values]	The results showed that the use of spiritual care can decrease depression in the intervention group.
Moghadam et al.	2021	Effect of Spiritual Care Program on Quality of Life in Patients with Heart Failure	Iran	Cardiova scular and Medical Research Centre	The present study aimed to determine the effect of a spiritual care program on QoL (psychological conditions such as depression) in patients with HF.	Randomized controlled trial	84 HF patients	1. Iranian Heart Failure Quality of Life (IHF-QoL)questionnaire (Naderi et al., 2012) 2. Spirituality questionnaire (Parsian and Dunning, 2009)	two virtual educational sessions- (each 1.5 h) as well as a 1-month follow-up three times a week for 1 h per session via WhatsApp	Spiritual interventions included strengthening and modifying the four dimensions of human communication (with God, others, self, and creation)	Dimensions of QoL in mental limitations (depression) and self-care were significantly improved in the intervention group, compared to the control group.
Masjedi- Arani et al.	2020	Effectiveness of An Islamic Approach to Hope Therapy on Hope, Depression, and Anxiety in Comparison with Conventional Hope Therapy in Patients with Coronary Heart Disease	Iran	Hospital	The purpose of this study was to investigate the effect of hope therapy on anxiety and depression using an Islamic approach and compare this approach with conventional hope therapy in coronary heart disease (CAD) patients.	Randomized trial	45 patients with CAD	The Hospital Anxiety and Depression Scale (HADS)- (Kaviani et al., 2009)	Eight 1.5- h sessions	Understanding stresses of cardiac diseases and stress relief strategies, self- calming training, regulating mood and thoughts, self- reflections practices.	Islamic and conventional hope therapy both significantly increase hope and decrease depression.
Nikrahan et al.	2016	Effects of Positive Psychology Interventions on Risk Biomarkers in Coronary Patients: A Randomized, Wait-List Controlled Pilot Trial	Iran	Hospital	Investigated the effect of 3 distinct PPIs on risk biomarkers in cardiac patients.	Randomized controlled trial	69 patients with recent CAB Gor percutaneous intervention	Beck Depression Inventory-II (Beck et al., 1996)	Three 6- week in- person PPIs (based on the work of Seligman, Lyubomirsky, or Fordyce)- each framework has spiritual intervention techniques	Expressing positive emotions, and making social connections actively focus on increasing optimism and gratitude, and realistic goals. Strengthening virtues, development, forgiveness, and spirituality, focusing on positive personality traits, enhancing meaning in life, flow, and mindfulness.	PPIs might be effective in reducing risk biomarkers as well as depression in high-risk cardiac patients
Ehsan etal.	2019	Comparison of the Effects of Islamic Spiritual Education and Breathing Techniques with Heart Rate Variability Feedback Therapies on Heart Rate Variability, Psychophysiologica l Coordination andStress in Patients Undergoing Coronary Artery Bypass Graft Surgery	Iran	Hospital	The current study aimed to compare the effect of Islamic education and breathing techniques with heart rate variability (HRV)biofeedback therapies on HRV, psychophysiology coordination, and stress in patients undergoing CABG	Quasi- experimental study	60 Patients	Depression Anxiety Stress Scale (Lovibond and Lovibond, 1995)	8 weeks (Two-h session per week)	Religious-based therapy using Islamic andQur'an teachings followed by doing home works and exercises	Results showed a significant difference among the three groups in the increase of HRV and psychophysiological coordination and decrease of stress

### 3.3 Culturally tailored intervention as therapeutic tools

One common aspect of the reviewed articles is the use of culture and spirituality in the treatment of mental health issues experienced by cardiac patients. Research conducted by Abdi et al. (2019), AbuRuz et al. (2023), Amjadian et al. (2020), Ehsan et al. (2019), and Masjedi-Arani et al. (2020) underscores the critical role of religious and spiritual factors in therapeutic practices. Specific techniques such as Islamic hope therapy, which instills a sense of optimism rooted in faith, the recitation of the Quran for its calming and reflective qualities, and coherence training aimed at fostering mental clarity and resilience have shown significant promise in alleviating psychological distress.

### 3.4 Reduction in depression and anxiety

The studies indicate that the various intervention programs help in reducing depression and anxiety levels in the participants. Tajbakhsh et al. (2018) and AbuRuz et al. (2023) reported a significant reduction in depression and anxiety in post-intervention findings. Similarly, Amjadian et al. (2020) and Ehsan et al. (2019), noted that religious and breathing interventions significantly reduced the depressive and anxious signs and symptoms. The results indicate that religious therapy demonstrated notably greater effectiveness in alleviating depression among patients compared to the effectiveness of breathing therapy for anxiety. These compelling findings underscore the importance of integrating spiritual and culturally sensitive approaches in the treatment of emotional distress, highlighting the need for holistic methods in mental health care.

### 3.5 Enhancement of positive psychological states

Various interventional approaches have significantly enhanced hope, life satisfaction, and spirituality in individuals. In a study conducted by Masjedi-Arani et al. (2020) therapies grounded in either Islamic principles or conventional hope practices were implemented, leading to notably higher scores of hope among participants. Meanwhile, research by Moghadam et al. (2021) emphasized the role of spirituality, demonstrating marked improvements in the quality of life (QOL) for those involved. These compelling results illustrate the effectiveness of culturally relevant coping strategies, which not only helps diminish negative emotions but also fosters uplifting psychological transformations and promotes better overall health and wellbeing.

### 3.6 Cultural beliefs and societal norms

Cultural beliefs and societal norms play a critical role in the effectiveness of interventions (Abdi et al., 2019; Amjadian et al., 2020). In collectivist societies, approaches that emphasize individual autonomy often faces resistance, making community-based strategies more effective (Moghadam et al., 2021; Nikrahan et al., 2016). Masjedi-Arani et al. (2020) and Tajbakhsh et al. (2018) highlighted gender roles, religious beliefs, and mental health stigma significantly impacts the willingness to seek help and the success of therapeutic methods. Additionally, cultural factors such as authority, family dynamics, and relationships influence how people engage with intervention programs (AbuRuz et al., 2023; Ehsan et al., 2019). Therefore, tailoring interventions to fit cultural beliefs and societal norms is essential for their success in diverse settings.

### 3.7 Holistic health improvements

The studies provide compelling evidence on chronic illnesses, highlighting the critical interplay between mental and physical health. Nikrahan et al. (2016) identified that using positive psychological interventions with a spiritual component led to decreased inflammatory biomarkers (hs-CRP) and better CARg. In the same way, Amjadian et al. (2020) and Ehsan et al. (2019) have shown higher psycho-physiological coherencies through breath and spiritual therapy. Such results present a comprehensive perspective on the effects of mental health interventions considered in both affective and somatic aspects, in a broad range of patient samples suffering from coronary artery disease, heart failure, and undergone through cardiac surgery. In particular, this suggests that these interventions could be implemented across various healthcare settings.

### 3.8 Mechanism of change

The interventions demonstrate effectiveness through several mechanisms, including the enhancement of emotional control, the promotion of spiritual coping strategies, and the improvement of psycho-physiological facilitation. Research indicates that breathing exercises and spiritual activities notably increase relaxation and mindfulness, thereby assisting individuals in effectively managing stress and anxiety (Amjadian et al., 2020; Ehsan et al., 2019). Additionally, spiritual care interventions have been shown to foster self-awareness and encourage proactive self-care behaviors, empowering individuals to gain a deeper understanding of their emotional and physical needs (Moghadam et al., 2021). These mechanisms provide critical insights into how these interventions achieve positive outcomes and enhance wellbeing.

## 4 Discussion

The results provide a thorough demographic and methodological overview of the included studies, highlighting several key aspects relevant to the understanding of interventions targeting patients with cardiac diseases in hospital settings. The reviewed studies provide valuable insights into effective interventions for patients with heart diseases, with research conducted from January 2015 to December 2024. The total sample size of 651 patients offer a moderate yet focused representation of this patient group. Among the eight studies analyzed, seven are from Iran and one is from Jordan, indicating that the research is rooted in the Middle Eastern context. These regional patterns may reflect specific cultural or healthcare system characteristics that were considered in interpreting and applying the findings of this research.

Furthermore, the methodological rigor of the studies is evident. Out of eight studies, six employed randomized controlled trials (RCTs), the most reliable method for assessing intervention effectiveness. RCTs enhance the validity of the conclusions by reducing bias and increasing the likelihood of true cause-and-effect relationships (Krauss, 2021). Whereas two was quasi-experimental, which is less rigorous due to the lack of randomization. However, it still offers useful insights into the efficacy of interventions in real-world settings (Diener et al., 2022). Overall, the methodological consistency and hospital-based focus of the reviewed studies provide a strong basis for understanding intervention outcomes in controlled healthcare environments.

The studies involved a patient population diagnosed with cardiac diseases. This diversity guarantees that the treatment approaches are implemented in a wide sphere of cardiac diseases. Study participants were mainly allocated randomly to experimental/intervention and control arms across six studies, consistent with methodological assertion characteristic of randomized controlled trials. The equal distribution of participants in the two studies enhances the methodological techniques making it easier to compare the groups. Randomization enhances the internal validity of the research including the following; random assignment reduces the possibilities of selection bias thus giving adequate evidence on the effects of interventions (Krauss, 2021). Of the eight reviewed studies, there were 4 that clearly described gender distribution. This suggests that cardiac diseases are more prevalent in males. The results are in line with previous studies (Walli-Attaei et al., 2020; Zhao, 2021; Wang et al., 2023).

All studies included depression, as an outcome measure, which was measured by self-report inventories. These are highly reliable and standardized tools, used in clinical and research practice to assess the degree of depression (Sisay et al., 2024; Jackson et al., 2022; Zhong et al., 2023; Zhao et al., 2021). Additionally, using clinician-administered tools or biomarker-based assessments supplements these findings and offers a more comprehensive evaluation of depressive symptomatology (Nikrahan et al., 2016). Integrating personalized treatment methods can lead to more favorable mental health outcomes for cardiac patients, illustrating the importance of culturally sensitive practices in healthcare settings (Goldfarb et al., 2024; Alkhaibari et al., 2023). Researchers have identified that culturally relevant strategies are not merely supplementary; they connect meaningfully with the participants' intrinsic beliefs and values (Wisuda et al., 2024). Promoting spiritual engagement and cultural understanding enhances patients' acceptance of treatment and encourages active participation in their recovery process. This alignment with clients' cultural or spiritual orientations is crucial for fostering a supportive therapeutic environment, as it can significantly boost overall effectiveness and success rates.

The reviewed studies demonstrate that practices like Islamic hope therapy, Quranic recitation, and religious coherence training are the core approach that makes the delivery of mental health interventions relevant to participants' belief systems (Sert et al., 2025). The focus on spirituality resonates with the structures such as cultural sensitivity or cultural respect as the idea is to address interventions supported by culture and have a better foundation of trust between the person and the therapy process. Consequently, the reduction in depression and anxiety is attributed to the effectiveness of culturally appropriate interventions. It is argued that depression and anxiety being pertinent mental health issues across the globe require faceted approaches for effective treatment. Tajbakhsh et al. (2018) and AbuRuz et al. (2023) argue that culturally embedded therapies improve clinical appearance as well as provide participants with comfort and acceptance. According to Ehsan et al. (2019), religious therapy is somewhat more efficacious when treating depression compared to breathing exercises. This highlights the importance of need-based approaches tailored while considering an individual's cultural identity.

Mental health interventions guide client processes toward the minimization of symptoms; however, current research suggests enhancing positive constructs, such as hope, life satisfaction, and spirituality. Masjedi-Arani et al. (2020) and Moghadam et al. (2021) demonstrated that psychological treatment approaches entrenched in cultural and spiritual practices foster growth and resilience by alleviating distress such as hope therapy involves focusing people on positive future outcomes despite the difficulties. Likewise, those who focus on spirituality bring participants back in touch with goals/missions and doers, which are typically worn down by mental health issues. That is why the attempts to minimize the level of distress are supplemented with attempts to maximize the level of positive experiences, as the fundamental framework of positive psychology suggests (Nikrahan et al., 2016).

Psychosocial approaches to culture-related mental and physical illnesses are now obvious in the research literature and the findings from the studies presented twofold effects of culturally tailored interventions. Such as Nikrahan et al. (2016) identified significantly lowered inflammatory biomarkers and better levels of glycemic control as the outcomes of mental health interventions. In cardiovascular diseases, it becomes possible to address psychological and psychophysical factors, as well as the resulting (Topuz and Topuz, 2024). Nonetheless, the applicability of such results concerning other samples involving patients with heart failure or those after CABG surgery underscores the potential of culturally grounded interventions for mitigating co-morbidity of health issues. These findings echo the needs to integrate mental health into overall health.

Through literature, it is evident that policymakers need to develop strategies as well as wellness programs that incorporate SBIs making it valuable approach in rehabilitation and healthcare management for chronic illnesses such as cardiac diseases, cancer, etc. It is essential to understand the mechanism of how intervention processes exert effects on psychological health. This provides critical insight for refining and optimizing its applicability. The mechanism that stands out is emotional regulation enlightening how practices such as mindfulness with breathing exercises enable clients to control the way they respond emotionally to stressors (Fugate et al., 2024). On the next level spiritual practices promote a sense of belonging to something greater than the self and although this is uplifting in normal circumstances, hence it is particularly beneficial in times of stress (de Brito Sena et al., 2021). Further, psycho-physiological coherence including breathing exercises shows that the interventions are bonding both mental health and body dynamism resulting in overall wellbeing encompassing emotional, spiritual, and physical aspects (Kellmann and Beckmann, 2024).

### 4.1 Limitations and areas for future research

The study focuses on the published articles on spiritual-based interventions in the reduction of depression in cardiac patients within the MENA population, using only four databases which may limit the generalizability of findings to other cultural or religious groups and geographical areas as 7 studies out of those included in the review have been conducted in Iran. Meta-analysis and registered systemic reviews should be conducted in the future to ensure broader databases and to critically appraise the included studies more stringently. Therefore, cross-sectional, longitudinal, follow-up, and well-designed randomized controlled trials of culturally specific interventions are required evidence to assess the time course, efficacy, and cost-effectiveness of these forms of intervention.

## 5 Conclusion

This scoping review highlights the importance of integrating spiritual principles into psychological interventions that align with the cultural and religious values of the MENA population, enhancing the acceptability and effectiveness of these approaches. Moreover, addressing depressive symptoms in cardiac patients can lead to better adherence to treatment, improved quality of life, and reduced healthcare costs, ultimately benefiting both patients and healthcare systems.
