# Supplementary material for: Exploring efficacy of spiritual-based interventions (SBIs) in addressing depressive symptoms among cardiac patients in MENA region: a scoping review
Source: Front Psychol. 2025 Apr 8;16:1552678. doi: 10.3389/fpsyg.2025.1552678 (PMC12012580; doi:10.3389/fpsyg.2025.1552678)
Supplement: Supplementary file 2 [file Table_2.docx]

Table 2. Critical appraisal of methodological quality of included studies (n=8)

| Randomized Control Trials (RCTs) | AbuRuz et al.  2023 | Amjadian et al., 2020 | Tajbakhsh et al., 2018 | Masjedi-Arani et al., 2020 | Moghadam et al., 2021 | Nikrahan et al.  2016 |
| --- | --- | --- | --- | --- | --- | --- |
| 1. Was true randomization used for the assignment of participants to treatment groups? | Y | U | Y | Y | Y | Y |
| 1. Was allocation to treatment groups concealed? | NA | NA | NA | NA | Y | NA |
| 1. Were treatment groups similar at the baseline? | Y | Y | Y | Y | Y | Y |
| 1. Were participants blind to treatment assignment? | Y | NA | N | NA | Y | Y |
| 1. Were those delivering treatment blind to treatment assignment? | NA | NA | N | NA | N | NA |
| 1. Were outcomes assessors blind to treatment assignment? | NA | NA | N | NA | NA | NA |
| 1. Were treatment groups treated identically other than the intervention of interest? | Y | Y | Y | Y | Y | Y |
| 1. Was follow up complete and if not, were differences between groups in terms of their follow up adequately described and analyzed? | Y | Y | Y | NA | Y | Y |
| 1. Were participants analyzed in the groups to which they were randomized? | Y | Y | Y | Y | Y | Y |
| 1. Were outcomes measured in the same way for treatment groups? | Y | Y | Y | Y | Y | Y |
| 1. Were outcomes measured in a reliable way? | Y | Y | Y | Y | Y | Y |
| 1. Was appropriate statistical analysis used? | Y | Y | Y | Y | Y | Y |
| 1. Was the trial design appropriate, and any deviations from the standard RCT design (individual randomization, parallel groups) accounted for in the conduct and analysis of the trial? | Y | Y | Y | Y | Y | Y |
|  | 100% | 88% | 75% | 100% | 91.6% | 100% |
| Quasi-Experimental Study | Abdi et al., 2019 | Ehsan et.al 2019 |  |  |  |  |
| 1. Is it clear in the study what is the ‘cause’ and what is the ‘effect’? | Y | Y |  |  |  |  |
| 1. Were the participants included in any comparisons similar? | Y | Y |  |  |  |  |
| 1. Were the participants included in any comparisons receiving similar treatment/care, other than the exposure or intervention of interest? | Y | Y |  |  |  |  |
| 1. Was there a control group? | Y | Y |  |  |  |  |
| 1. Were there multiple measurements of the outcome both pre and post the intervention/exposure? | Y | Y |  |  |  |  |
| 1. Was follow up complete and if not, were differences between groups in terms of their follow up adequately described and analyzed? | NA | NA |  |  |  |  |
| 1. Were the outcomes of participants included in any comparisons measured in the same way? | Y | Y |  |  |  |  |
| 1. Were outcomes measured in a reliable way? | Y | Y |  |  |  |  |
| 1. Was appropriate statistical analysis used? | Y | Y |  |  |  |  |
|  | 100% | 85% |  |  |  |  |
